# Heterologous expression and activity verification of ornithine decarboxylase from a wild strain of *Shewanella xiamenensis*

**DOI:** 10.3389/fmicb.2022.1100889

**Published:** 2022-12-20

**Authors:** Chang Liu, Guiyuan Wang, Xiangning Han, Limin Cao, Kaiqiang Wang, Hong Lin, Jianxin Sui

**Affiliations:** College of Food Science and Engineering, Ocean University of China, Qingdao, Shandong, China

**Keywords:** *Shewanella xiamenensis*, ornithine decarboxylase, heterologous expression, HPLC, enzymatic activity

## Abstract

*Shewanella xiamenensis* is widely found in spoilage fish, shrimp and other seafoods. Under suitable conditions, ornithine can be synthesized into putrescine, which may spoil food or endanger health. Our research used a wild strain of *Shewanella xiamenensis* isolated from “Yi Lu Xian” salted fish (a salting method for sea bass) as a research object. According to the database of National Center of Biotechnology Information (NCBI), the target ornithine decarboxylase (ODC) gene SpeF was successfully amplified using the wild strain of *Shewanella xiamenensis* as the template. Sequencing alignment showed that the SpeF of the wild strain had more than 98% homology compared with the standard strain. The amino acid substitution occurred in the residues of 343, 618, 705, and 708 in the wild strain. After optimizing the expression conditions, a heterologous expression system of ODC was constructed to achieve a high yield of expression. The amount of 253.38 mg of ODC per liter of LB broth was finally expressed. High performance liquid chromatography (HPLC) and subsequent ODC activity verification experiments showed that hetero-expressed ODC showed a certain enzyme activity for about 11.91 ± 0.38 U/mg. Our study gives a new way to the development of a low-cost and high-yield strategy to produce ODC, providing experimental materials for further research and elimination of putrescine in food hazards.

## 1 Introduction

Putrescine is one of the most common biogenic amines that has many physiological activities, such as promoting the formation of biofilms (Bjornsdottir-Butler et al., 2018; Bao et al., 2021), affecting cell growth (Bogdanović et al., 2020) and the synthesis of ferro-carriers (Bujnakova et al., 2014; Bustin et al., 2020), and is commonly found in meat, fermented products and seafood (Costantini et al., 2013; Chmiel et al., 2020; Cagiada et al., 2021). More importantly, a high concentration of putrescine not only affects the sensory characteristics of food, but also causes toxicological reactions. Putrescine can enhance the toxic effect of histamine, causing headaches, nausea, vomiting, and other adverse reactions (Huang et al., 2015; Hofmann et al., 2020; Fu et al., 2021). Therefore, putrescine is an important spoilage indicator in food (Kanjee et al., 2011).

Putrescine in food is mainly produced by specific microorganisms (Luqman, 2012; Lotfi et al., 2017; Mühlmann et al., 2017; Li et al., 2021), and is produced mainly in two pathways, including the ornithine decarboxylation (ODC) pathway and the arginine decarboxylation (ADC) pathway (Önal et al., 2013; Nakamura et al., 2019; Omer et al., 2021). In the study of putrescine production by *Chlamydomonas*, the ODC activity is almost 5 times than that in ADC activity, and is, undoubtedly, the main pathway of putrescine synthesis (Ramos-Molina et al., 2015; Rio et al., 2018). In the ODC pathway, three synthases including SpeC, SpeF, and PotE are involved in the putrescine production. Among them, it is found that the SpeF of ornithine into putrescine is widely regarded as the main pathway of putrescine production. Therefore, putrescine formation could be affected by inhibiting the synthesis or supply of ornithine (Rio et al., 2018, 2019). Although it has been shown that ODC produced by microbial systems converts ornithine to putrescine, there is no study on the ODC acting *in vitro*.

The wild strain of *Shewanella xiamenensis* used in this study was isolated from the “Yi Lu Xian” salting process in our previous research. According to our previous research, this wild strain of *Shewanella xiamenensis* could produce putrescine in the presence of only glucose, ornithine, and NaCl. The ODC system responsible for putrescine production in *Shewanella xiamenensis* remained unknown. Therefore, the SpeF gene of this wild strain was selected for producing the heterologous ODC, and the target gene was amplified by using the designed specific primers. The heterologous expression system was optimized to achieve a high yield of ODC expression. Hetero-expressed ODC was then used to verify its ODC activity by High performance liquid chromatography (HPLC) and other experiments *in vitro*. We hope this study gives a new way to the development of a low-cost and high-yield strategy to produce putrescine for further investigation, contributes to a broader understanding of the production mechanism of biogenic amines in the food system and provides theoretical support for effectively ensuring the safety of seafood.

## 2 Materials and methods

### 2.1 Materials and instruments

BL21 (DE3) competent cells, plasmid pET-30a, and specific primers were synthesized and purchased in Beijing Genomics institution Co., Ltd. (China). Eight biogenic amines standards and dansulfonyl chloride were purchased from Sigma Co., Ltd. (USA). Methanol and acetonitrile (Chromatographic use) was purchased from Tedia Co., Ltd. (USA). Broad-spectrum protein Marker (11–180 KDa), L (+) -Ornithine hydrochloride, pyridoxal 5-phosphatemonohydrate and 5X protein loading buffer were purchased from Solarbio Co., Ltd. (China).

### 2.2 Gene synthesis and the construction of recombinant strains

The gene sequence of standard strain of *Shewanella xiamenensis* (Gene ID: 75190569), used for the comparison with the wild strain of *Shewanella xiamenensis* in our research, was obtained from the National Center of Biotechnology Information (NCBI) database (Romano et al., 2012). The specific primers for amplifying the speF gene were designed. Single colonies of the experimental strain were selected and picked up in sterile ultrapure water and heated at 100^°^C for 15 min to obtain the complete genome of the experimental strain. The obtained whole genome was used as the DNA template, and the target gene SpeF with the cloned gene size of 2,163 bp was amplified by designing the specific primers, including primer-forward (5′-TAA TTG GGT GGG CAG CAT-3′) and primer-reverse (5′-TGC ACC ATC CAG CTT ACT CA-3′) (Sakamoto et al., 2012). PCR conditions were set as follows: 95^°^C for 5 min, later, 95^°^C for 15 s, 55^°^C for 15 s, and 72^°^C for 30 s for 35 cycles and finally extension at 72^°^C for 5 min. Nucleic acid electrophoresis was used to isolate PCR products. Later, the PCR products were sent to Tsingke company (Qingdao, China) for further sequencing and analyzing. The pET-30a expression vector with 6 × His tag was used for the heterologous expression. After analyzing the gene sequencing, *Sac*I (5′-GAGCT′C-3′), and *Eco*RI (5′-G′AATTC-3′) were selected as restriction sites. The target gene was cloned by the method of double enzyme digestion and T4 ligase to construct the recombinant plasmid.

The recombinant plasmid was then transferred to BL21 competent by thermal transformation. In brief, 10 μL of plasmid was added to 100 μL of competent cells. After standing on ice for 30 min, the transform system was treated in a water bath with 42^°^C for 45 s and placed on ice for another 2 min. Later, 1 mL of LB liquid medium was added and cultured at 37^°^C for 60 min with shaking at 220 rpm. The components were then plated on LB agar plates containing 50 μL/mL kanamycin for growing at 37^°^C for 16 h. Clones were randomly selected from the plate for further culturing, plasmid extraction, and sequencing. Clones with correct sequencing results were identified as positive clones and stored for the further expression use.

### 2.3 Expression and purification of recombinant ODC

Freshly transformed *E. coli* BL21 cells were inoculated into 10 mL of LB broth containing 50 μg/mL kanamycin and cultured overnight (16 h) at 37^°^C with shaking at 200 rpm. Afterward, the 5 mL of medium was transformed into 1,000 mL of LB broth containing 50 μg/mL kanamycin and cultured at 37^°^C for 4 h. Different induced temperatures, culturing time and concentrations of isopropyl-β-D-thiogalactoside (IPTG) were investigated for the best condition of heterologous expression (Santiyanont et al., 2019). The cells were obtained by centrifugation at 4,000 rpm for 30 min and then resuspended in PBS buffer at 4^°^C (0.01 M, pH 7.4). After breaking by a low-temperature high-pressure cell crusher for 10 min, the soluble protein containing 6 × His-tag was then separated by the centrifugation at 4^°^C and 13,000 rpm for 20 min.

The nickel immobilized metal ion affinity chromatography (IMAC) Ni-NTA resin column was used for the purification of recombinant protein (Smart-lifesciences, China). After washing by binding buffer (50 mmol/L Tris, 500 mmol/L NaCl, pH 8.0), the soluble protein containing 6 × His-tag was loaded onto the column. After collecting the flow-through sample, the column was washed with a fivefold column volume of PBS (0.01 M, pH 7.4) to remove the non-specific binding proteins. Later, the column was washed with the binding buffer containing 10, 20, 50, 100, and 200 mM of imidazole, and the recombinant protein was then eluted by the binding buffer containing 500 mM imidazole. Finally, the eluted protein was dialyzed by the binding buffer at 4^°^C and changed every 8 h and repeated three times to remove the imidazole.

### 2.4 SDS-PAGE of purified ODC

Pre-fabricated protein electrophoresis gel was used for SDS-PAGE verification. The protein samples were mixed with 5 × protein loading buffer and heated with boiling water for 7 min. After electrophoresis under the voltage of 120 V for about 60 min, the G250 gel solution and the decolorizing solution (methanol, acetic acid and ultrapure water with a ratio of 1:12:17) were used for dyeing and decolorization of gel to observe the electrophoresis bands, respectively.

### 2.5 Determination of ODC activity

#### 2.5.1 Preliminary treatment

The L (+)—ornithine hydrochloride and Pyridoxal 5-phosphatemonohydrate were used as a substrate and a coenzyme to determine the activity of ODC, respectively. The reaction solution (2,000 μL) was prepared containing 1,500 μL Hepes buffer solution (10 mM, pH 6.8–8.0) of dissolved L (+)—ornithine hydrochloride (10 mM) and pyridoxal 5-phosphatemonohydrate (1 mM) and 500 μL enzyme solution. The whole reaction process was conducted in a sterile environment and all solutions were treated with 0.22 μm sterile membranes. After incubation at 37^°^C for 4 h, the reaction was terminated by adding 2 mL of 5% (v/v) TCA. The mixture was centrifuged at 8,000 rpm for 20 min. A control group (No ODC added) was used to eliminate the effect of environmental implications. The supernatant was collected for further HPLC determination.

#### 2.5.2 High performance liquid chromatography (HPLC)

The putrescine needs to be derived before the determination of HPLC since it has no UV absorption peak. The method of derivation and determination of putrescine was referred to Fu’s research (Soleymani and Mostafaie, 2019). In brief, 1 mL of saturated NaHCO_3_ was added into the supernatant, adjusting the pH value to 9.0 by using the 1 M NaOH. Then, 1 mL of dansulfonyl chloride (10 mg/mL) was added and mixed in a water bath at 60^°^C for 20 min. After that, 3 mL of ether was added for extraction. After shaking and standing for 10 min, the upper organic phase was transferred and dried by nitrogen. Later, 0.9 ml of acetonitrile and 0.1 mL of ammonia were added for re-dissolution, followed by passing through a 0.22 μm filter membrane. By comparing the chromatogram with the standard product, the type of amines produced by amine-producing was determined. During the HPLC detection, acetonitrile and ultra-pure water were used as the mobile phase A and mobile phase B. C18 column (5 μm × 250 mm × 4.6 mm) was used for detection under the temperature of 40^°^C and the detection wavelength of 254 nm. The flow rate and the sample size were set at 1 mL/min and 20 μL, respectively. Eight bioamine standard solutions were mixed with the concentrations of 0, 0.2, 1, 5, 10, and 20 mg/L for subsequent derivative steps and bioamine standard curve comparison.

#### 2.5.3 The calculation of ODC activity

The enzyme activity is defined as the amount of substrate converted into product per minute per milligram of the protein, as shown in the following formula. Protein concentration in the following formula was determined by Bradford’s method.


E⁢n⁢z⁢y⁢m⁢a⁢t⁢i⁢c⁢a⁢c⁢t⁢i⁢v⁢i⁢t⁢y=

$$\frac{T\,h\,e\,m\,o\,l\,a\,r\,a\,m\,o\,u\,n\,t\,o\,f\,t\,h\,e\,s\,u\,b\,s\,t\,r\,a\,t\,e\,c\,o\,n\,v\,e\,r\,t\,e\,d\,i\,n\,t\,o\,p\,r\,u\,d\,u\,c\,t\,\left(m\,o\,l\right)}{T\,h\,e\,w\,e\,i\,g\,h\,t\,o\,f\,p\,r\,o\,t\,e\,i\,n\,\left(m\,g\right)\times R\,e\,a\,c\,t\,i\,o\,n\,t\,i\,m\,e\,\left(m\,i\,n\right)}$$

### 2.6 Data analysis

All data in this study were processed by SPSS (Version 20.0; SPSS Inc., Chicago, USA). Comparisons were made using a one-way analysis of variance (ANOVA). *p* < 0.05 was considered a statistically significant difference. Duncan’s test was used to evaluate the statistical significance of differences. OriginLab (Version 8.1; OriginLab Inc., Massachusetts, USA) and Adobe Photoshop (Version 19.1.3; Adobe Systems Incorporated Inc., USA) were used for data processing, spectrum analysis, and chart making.

## 3 Results and discussion

### 3.1 The cloning of ODC coding sequence

**FIGURE 1:**
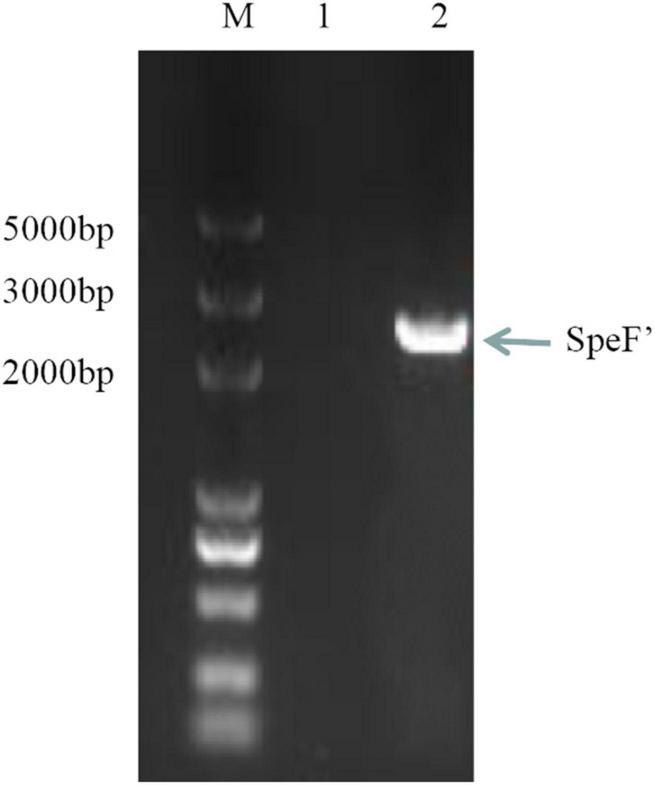
SpeF amplicon obtained from the polymerase chain reaction. Lane M: DNA Marker (5,000 bp). Lane 1: negative control (no DNA template added). Lane 2: SpeF amplicon gene from the wild strain of *Shewanella xiamenensis*.

**FIGURE 2:**
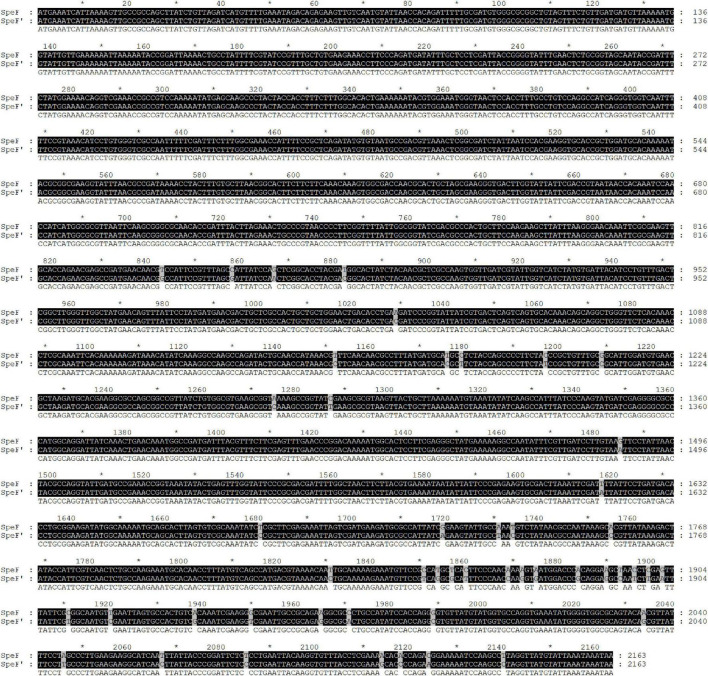
Nucleotide sequence alignment of PCR product amplified by a wild strain of *Shewanella xiamenensis*, which was named as SpeF’, and ornithine decarboxylation gene from NCBI database, which was named as SpeF.

**FIGURE 3:**
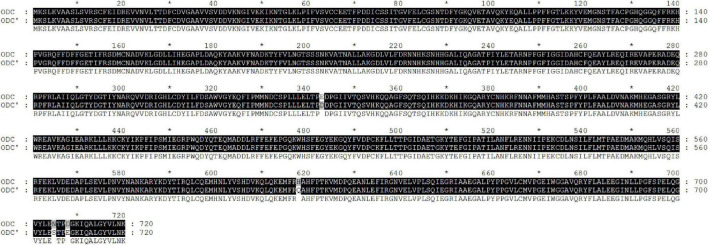
Amino acid sequence alignment of PCR product amplified by a wild strain of *Shewanella xiamenensis*, which was named as ODC’, and ornithine decarboxylation gene from NCBI database, named as ODC.

The standard ODC sequence is available with the gene ID 58507711 in the NCBI database, with the predicted molecular mass and pI value of the ODC being 80 kDa and 5.81, respectively. As shown in Figure 1, the ODC encoding gene SpeF from the wild strain was successfully amplified by PCR reaction using the designed primers, while the target band was bright, with a molecular band between 2,000 and 3,000 bp. After sequence analyzing and comparing with the speF gene of standard *Shewanella xiamenensis* on NCBI, the similarity between PCR product and SpeF gene was 98%, with 48 bases different from the standard speF gene sequence, as shown in Figure 2. After converting into the amino acid sequences, the similarity between ODC from wild strain and standard ODC was about 99%. Four amino acid sites were substituted with ODC in the standard strain (Figure 3). Glutamate was replaced by aspartic acid at residue 343, glutamine was replaced by histidine at residue 618, asparagine was replaced by serine at position 705, and aspartic acid was replaced with glutamic acid at residue 708, which might affect the type and arrangement of amino acids, protein structure and activity (Taylor and Eitenmiller, 1986; Tassoni et al., 2018; Sunde et al., 2021). However, from the perspective of the characteristics of amino acids, the properties of these substituting amino acids were similar compared to the original amino acids, except for the glutamine replaced by histidine at residue 618. ExPasy was used to predict the properties of the expressed protein. According to the prediction results, the molecular weight of the expressed protein was 81.064 kDa, with a theoretical pI value of 5.81, which showed a high similarity to standard ODC. The ExPasy also provided some other theoretical information about the protein, including 88 negatively charged residues (Asp + Glu) and 74 positively charged residues (Arg + Lys). The instability index (II) was 37.35, indicating that the protein was relatively stable. The aliphatic index and grand average of hydropathicity (GRAVY) were 82.50 and 0.230, respectively. Therefore, it was considered that these four sites were not able enough to affect the activity of ODC. Later, the ODC sequence of wild strain was considered to be introduced into the expression plasmid pET-30a for further heterologous expression.

### 3.2 Heterologous expression and purification of ODC

**FIGURE 4:**
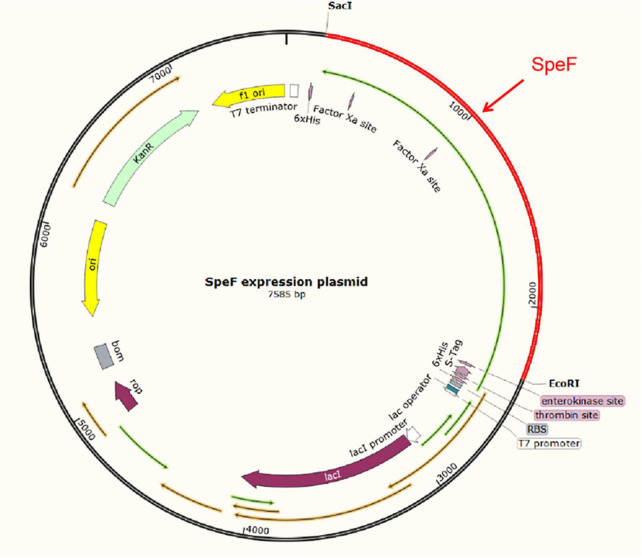
The schematic diagram of the pET-30a-SpeF recombinant plasmid. The *Sac*I and *Eco*RI were selected as restriction sites.

**FIGURE 5:**
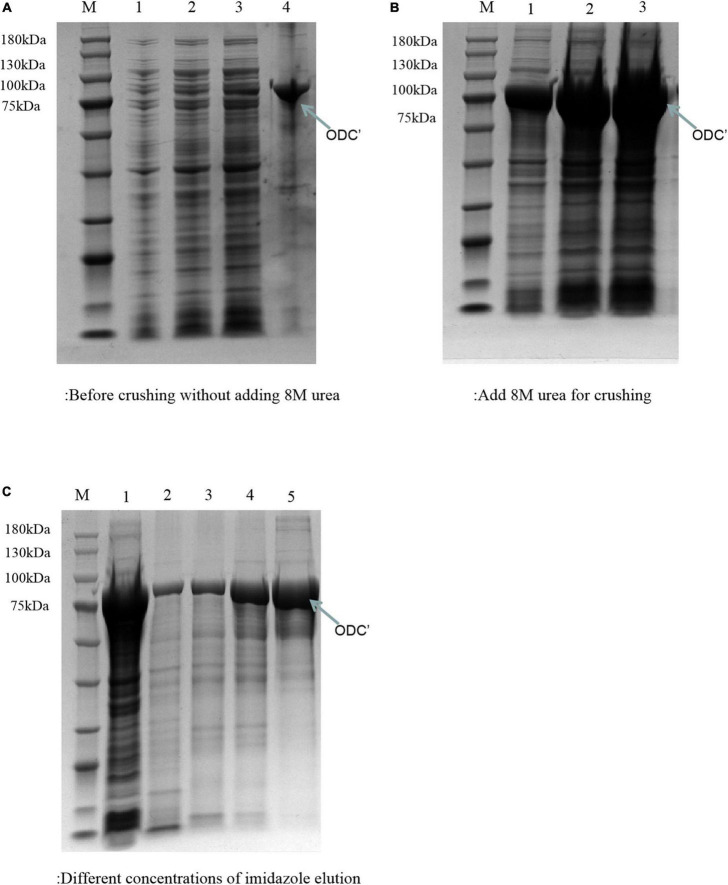
SDS-PAGE analysis of ODC under the express condition of 1 M IPTG at 25^°^C for 12 h. M represented the protein marker (180 kDa). **(A)** Represented the components before adding 8 M urea. Lanes 1–3 represented the supernatant of crushed cells centrifuge precipitate with different volumes (1, 3, and 5 μL), respectively. Lane 4 represented the precipitate of crushed cells. **(B)** Represented the components after adding 8 M urea. Lanes 1–3 represented the precipitated solutions with different volumes (1 μL, 3 μL, 5 μL), respectively. **(C)** SDS-PAGE verification of the different concentrations of imidazole eluents. Lane 1 represented the supernatant after breaking by a low-temperature high-pressure cell crusher. Lanes 2–5 represented the eluents of different concentrations of imidazole (20, 50, 100, and 200 mM), respectively.

The theoretical plasmid diagram is shown in Figure 4. After the recombinant plasmid was successfully constructed and introduced into the BL21 competent cells, the positive clone with correct sequence results was used for subsequent heterologous expression experiments. At first, a high final concentration of IPTG (1 M), culturing at 25^°^C for 12 h was used as the induction condition to express the ODC protein. SDS-PAGE result showed that the expressed proteins existed in the precipitation as inclusion bodies (Figures 5A, B). This may be because the molecular weight of expressed ODC is about 80 kDa, macromolecular protein usually requires lower temperature and a longer time to complete its structure, and the IPTG with too much high concentration is not conducive to the structural integrity of ODC (Valle et al., 2020; Wang et al., 2020). Therefore, a high urea concentration was used to dissolve the protein after being purified by Ni-NTA resin column with gradient concentrations of imidazole. The SDS-PAGE result showed that the target protein band was bright with a correct molecular weight of 80 kDa (Figure 5C), which was consistent with the previously reported ODC size (Wang et al., 2016). However, after the renaturation with arginine and glutamine, the renatured protein did not show the apparent activity of ODC. This may be due to the fact that although the molecular weight of the expressed protein is correct, the two subunits that play an essential role may not be fully renatured to the complete structure and show their ODC activity through the existing renaturation methods (Zare and Ghazali, 2017).

**FIGURE 6:**
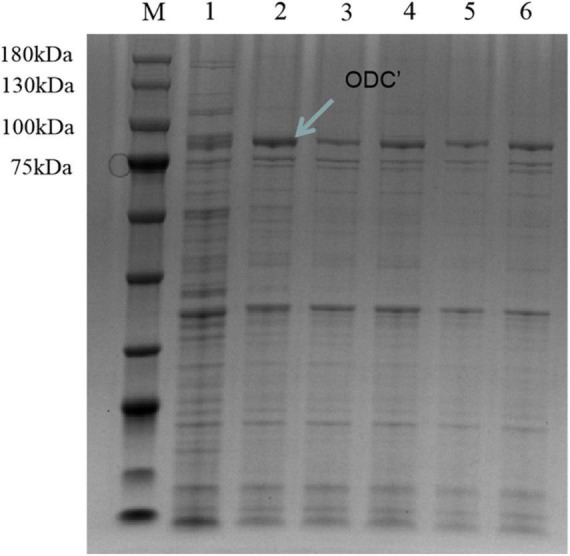
SDS-PAGE verification of hetero-expression ODC under the inducement condition at 20^°^C for 14 h. M represented the protein marker (180 kDa). Line 1 represented hetero-expression ODC with no inducement. Lines 2–6 represented the hetero-expression ODC induced with 0.05, 0.1, 0.3, 0.5, and 0.7 mM IPTG, respectively.

**FIGURE 7:**
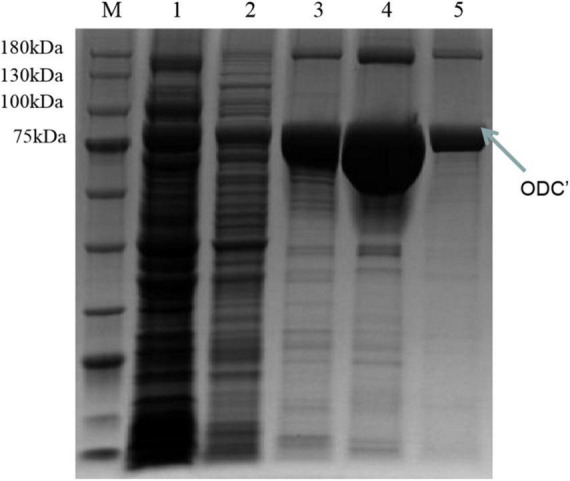
SDS-PAGE verification of the different concentrations of imidazole eluents after the inducement of at 20^°^C for 14 h with the final concentration IPTG of 0.05 mM. M represented the protein marker (180 kDa). Lane 1 represented the supernatant after breaking by a low-temperature high-pressure cell crusher. Lanes 2–5 represented the eluents of different concentrations of imidazole (20 mM, 50 mM, 100 mM, 200 mM), respectively.

Therefore, the induced expression strategy of the ODC protein was changed. The inducement conditions of 20^°^C for 14 h was selected by evaluating the effect of different IPTG concentration (0, 0.05, 0.1, 0.3, 0.5, and 0.7 mM) on protein expression. As shown in Figure 6, SDS-PAGE results showed that the target protein band was most obvious under the induction condition of the final concentration of 0.05 mM IPTG, indicating the best induction condition of ODC protein expression. As shown in Figure 7, after being purified by Ni-NTA resin column with gradient concentrations of imidazole, the recombinant ODC eluted by 200 mmol/L imidazoles showed an obvious band on SDS-PAGE verification with the molecular weight of 80 kDa. The protein was freeze-dried to calculate the expression yield and the result showed that 253.8 ± 2.4 mg ODC protein could be obtained per liter of LB broth.

### 3.3 The determination of ODC activity

**FIGURE 8:**
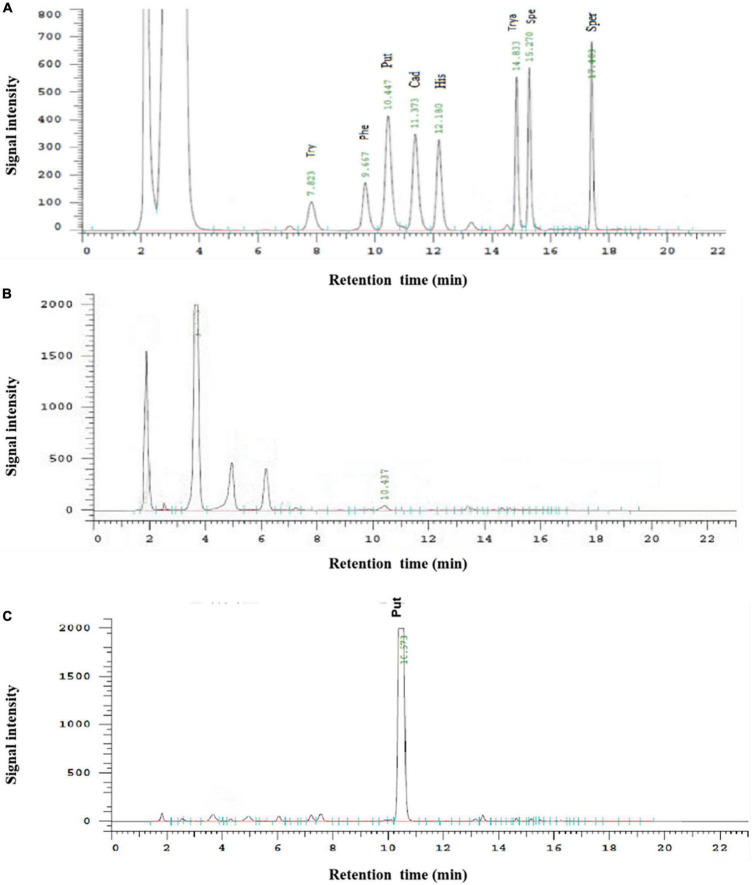
The HPLC determination of biogenic amines. **(A)** Represented the HPLC determination result of the mixture of eight biogenic amines standards. **(B)** Represented the HPLC determination result of the negative control. **(C)** Represented the HPLC determination result of hetero-expressed ODC.

The determination of putrescine by HPLC has the advantages of simplicity, rapidity, high sensitivity, and good reproducibility (Zhang et al., 2019). As shown in Figure 8A, all eight biogenic amines standards could be separated well within 20 min, and each peak was symmetrical without baseline drift during the HPLC determination. Therefore, this method can be used for the quantitative analysis of putrescine. As can be seen from Figures 8B, C, there was only one peak of putrescine and no peak of other biogenic amines in the liquid chromatography in the experimental group while the negative group without ODC showed no significant bioamine peaks. After calculating, the standard curve of putrescine is y = 35753x-4057.3 (*R*^2^ > 99.9%), which showed the high accuracy and good repeatability, and could be used for quantitative analysis of putrescine content. By comparing the standard curve, it can be calculated that the expressed protein had a certain specific activity of ODC and could be quantified to 11.91 ± 0.38 U/mg, indicating that the expressed protein in our study has the ability to convert ornithine into putrescine.

## 4 Conclusion

In this study, the SpeF gene encoding ODC isolated from a wild strain was successfully amplified. By comparing to the NCBI database, SpeF showed high similarity of standard SpeF with four amino acid differences compared to the standard strain. After constructing and optimizing the heterologous expression system, the final concentration of 0.05 mM IPTG at 20^°^C for 14 h was selected for the best inducement condition. The ODC was expressed with a high yield of 253.8 ± 2.4 mg per liter of fermentation broth with a molecular weight of about 80 kDa, which was consistent with the previously reported ODC size. The HPLC determination and further experiments demonstrated that the hetero-expressed ODC in our research could convert L-ornithine to putrescine in a buffer system containing only L-ornithine and coenzyme with an enzyme activity of 11.91 ± 0.38 U/mg.

## Data availability statement

The data presented in this study are deposited in the NCBI repository, accession number: OP985534.

## Author contributions

CL and GW wrote the manuscript and made the figures under the supervision and revision of XH, LC, KW, HL, and JS. All authors contributed to the article and approved the submitted version.
